# Supplementary material for: Foreign body responses in mouse central nervous system mimic natural wound responses and alter biomaterial functions
Source: Nat Commun. 2020 Dec 4;11:6203. doi: 10.1038/s41467-020-19906-3 (PMC7718896; doi:10.1038/s41467-020-19906-3)
Supplement: Supplementary file 1 — Supplementary Information [file 41467_2020_19906_MOESM1_ESM.pdf]

Supplementary Information

Foreign body responses in mouse central nervous system mimic natural wound responses and alter biomaterial functions

Timothy M. O'Shea[1], Alexander L. Wollenberg[2], Jae Kim[1], Yan Ao[1], Timothy J. Deming[2,3], and Michael V. Sofroniew[1]

[1] Department of Neurobiology, David Geffen School of Medicine, University of California, Los Angeles, California 90095-1763, USA.

[2] Department of Chemistry and Biochemistry, University of California Los Angeles, Los Angeles, California 90095-1600, USA.

[3] Department of Bioengineering, University of California Los Angeles, Los Angeles, California 90095-1600, USA.

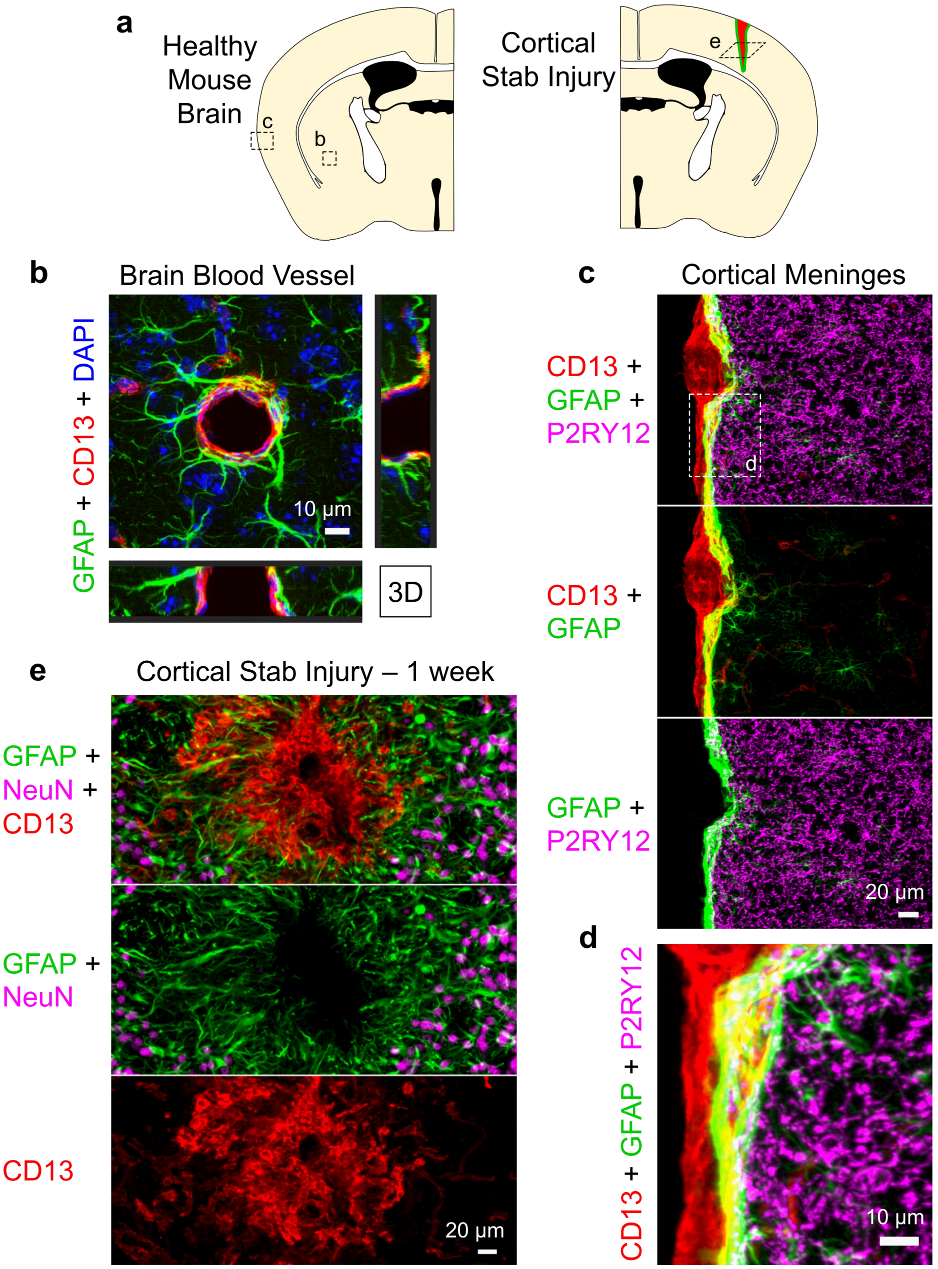

Supplementary Figure 1 | Non-neural cells express CD13 in both the healthy and injured mouse brain and interact with border-forming astrocytes. a. Schematics of healthy and stab injured mouse forebrain indicating locations of immunohistochemistry (IHC) images in b-e. **b.** Detail image of cerebrovasculature in mouse caudate putamen. CD13-positive pericytes along blood vessels are surrounded by cellular processes of GFAP-positive astrocytes which form glial limitans borders that segregate neural from non-neural tissue. **c. & d.** Survey and detail images of the outer surface of mouse cortex. CD13-positive stromal cells of the cerebral meninges are continually abutted by cellular processes of GFAP-positive astrocytes which form glial limitans borders that segregate neural from non-neural tissue around the entire CNS. P2RY12 staining shows microglia in healthy neural tissue. Overlapping and intertwined processes of stromal cells (red) and astrocytes (green) is seen as yellow. **e.** Detail image of a stab injury lesion in the cerebral cortex at one week after injury. CD13-positive cells accumulate at the lesion epicenter and are surrounded by the cellular processes of GFAP-positive astrocytes which segregate the CD13 non-neural compartment away from the immediately adjacent viable neural tissue compartment that contains NeuN-positive neurons.

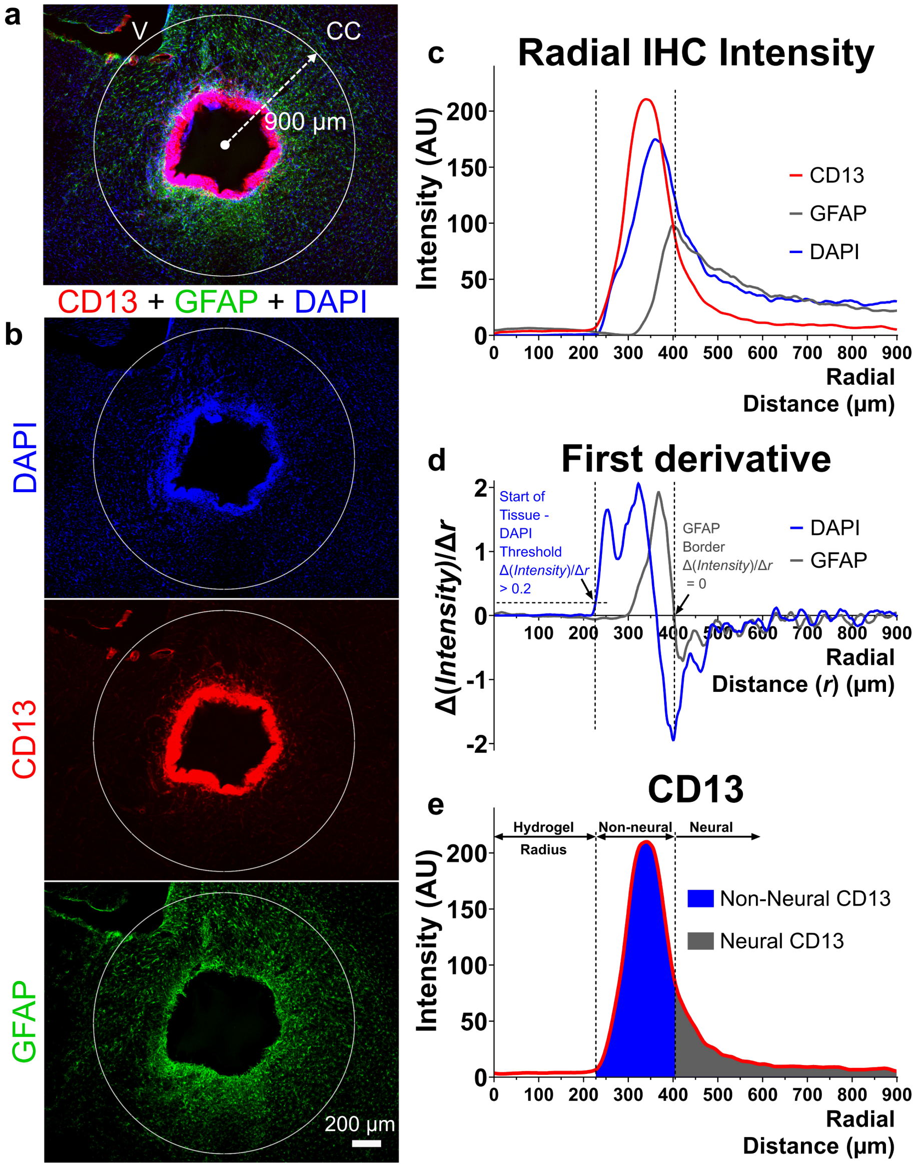

Supplementary Figure 2 | Quantification procedure used to characterize the CNS FBR to different hydrogel formulations. a. & b. Survey images of CD13-, GFAP- and DAPI-positive cells involved in the FBR to an example hydrogel. Hydrogel deposits were located within the caudate putamen (CP) with the ventricles (V) and corpus collosum (CC) on either side. A circular zone of analysis defined by an outer limit radius of 900 µm from the center of the hydrogel deposit was used for all samples. The zone of analysis encompassed the entire region of CP tissue around the hydrogels. **c.** Plots of average fluorescent signal intensity for the immunohistochemical (IHC) stains as a function of radial distance from the center of the hydrogel deposit. The average intensity for a specific IHC stain at a given radial distance, *r*, from the hydrogel deposit center was determined by averaging the intensity across the entire circumference of a circle with radius *r*. The average intensity was computed across the full range from 0 to 900 µm with sequential radius values increasing by a value of one pixel , where one pixel equated to 1.063 µm. **d.** Numerical differentiation methods were used to caculate the first derivative of the average IHC intensity plot for each IHC stain ($\Delta Intensity/\Delta r$), where *r* is the radial distance from the center of the hydrogel. The radial location of the host tissue/hydrogel interface, and hence the hydrogel radius, was defined as the radius value at which the first derivative of the DAPI intensity reached a fixed threshold of 0.2. This threshold value represented approximately 10% of the max first derivative value and was deemed to be a suitable value for detecting the start of DAPI-positive cells across a random assortment of hydrogels. The radial location of the astrocyte border was determined as the radial value where the first derviative of the GFAP intensity was zero, equating to the maxium value on the GFAP intensity plot. **e.** Area under the curve (AUC) (or definite numerical integral across a fixed range) calculations for the IHC average intensity plots were used to determine total values for either CD13 or GFAP. For CD13, the AUC value across the range between the hydrogel/tissue interface to the astrocyte border was defined as the total non-neural CD13 value while the CD13 intensity from the astrocyte border to the edge of the analysis region was defined as the neural tissue CD13 value. For total GFAP, a range from the hydrogel-tissue interface to the edge of the analysis region was assessed.

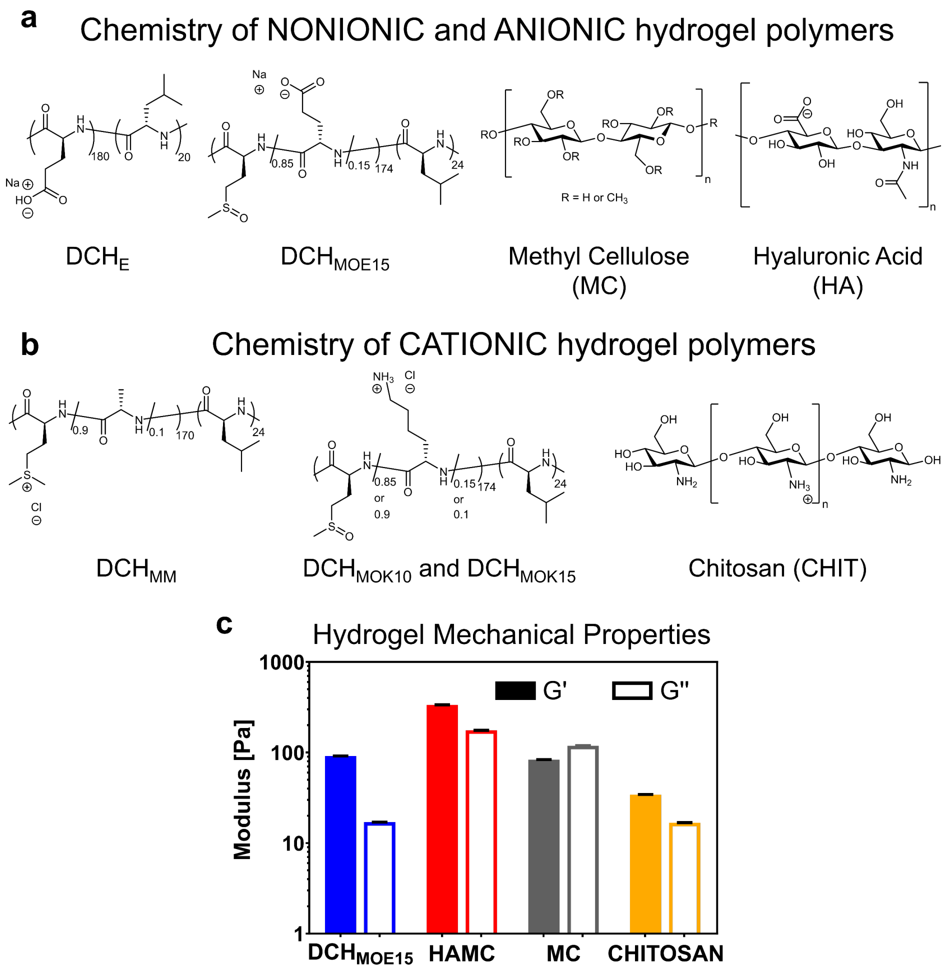

Supplementary Figure 3 | Chemical structures of different polymers used to formulate hydrogels evaluated in this study. a. Non-ionic and anionic polymers **b.** Cationic polymers. Note that DCH_{MOE15}, DCH_{MOK10} and DCH_{MOK15} consisted of DCH_{MO} to which small amounts of either negatively charged glutamate (E), or positively charged lysine (K) had been incorporated into the hydrophilic polypeptide block as a statistical copolymer. We also modified the poly(L-methionine-*stat*-L-alanine) segments in the parent polymer used to formulate DCH_{MO} by selective methylation of methionine residues to form the cationic methyl-sulfonium based DCH_{MM} [1]. **c.** Mechanical properties of some of the hydrogels used in this study as evaluated by dynamic rheology. Graphs are mean ± s.e.m with repeat measures per material being; n=60 for DCH_{MOE15}, n= 240 for HAMC, n= 196 for MC, n=240 for Chitosan. Mechanical properties of the other DCH used are detailed in our previous publications [1-4].

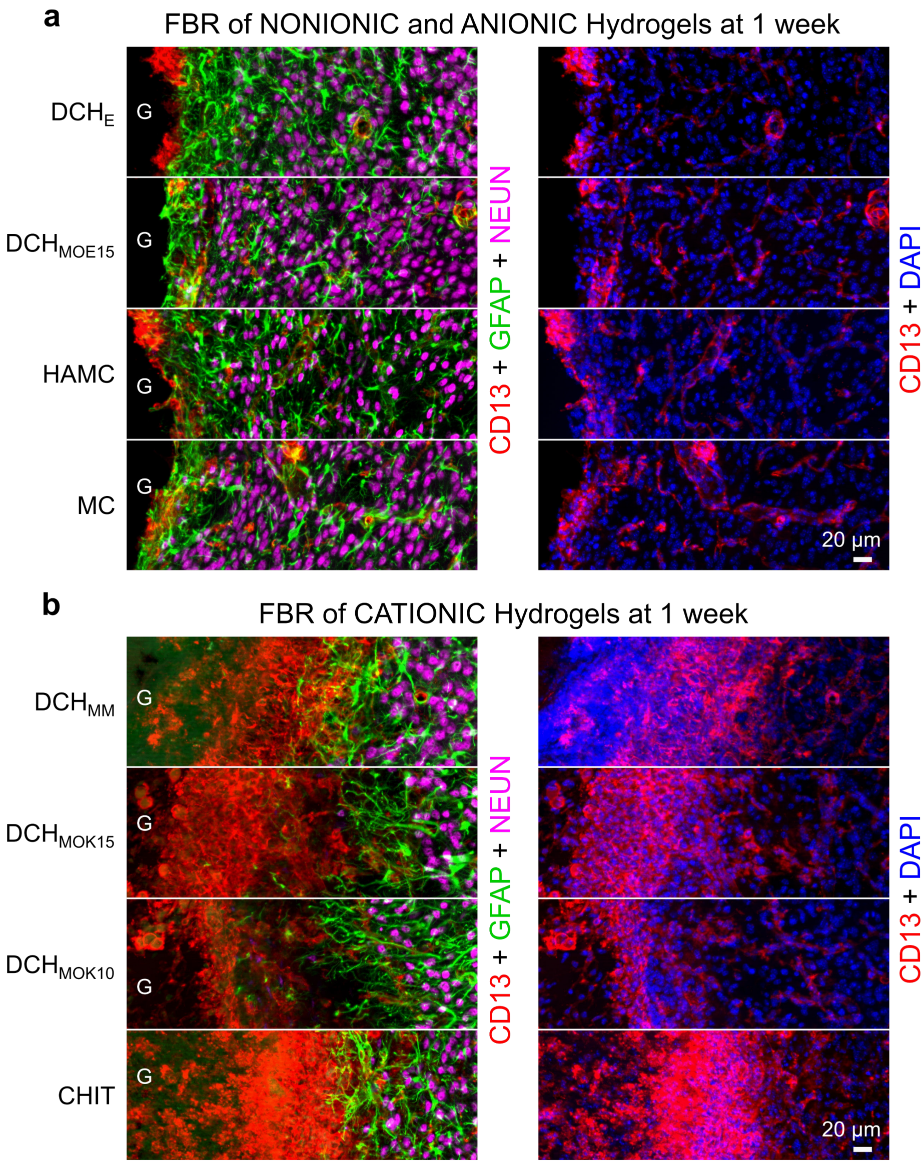

Supplementary Figure 4 | Detail images of different hydrogel FBRs at one week after injection into mouse caudate putamen (CP). a. Nonionic and anionic hydrogels exhibit barely detectable levels of CD13-positive cells at host interfaces. **b.** Cationic hydrogels exhibit large rims of CD13-positive cells infiltrating into deposits at their interfaces with host tissue.

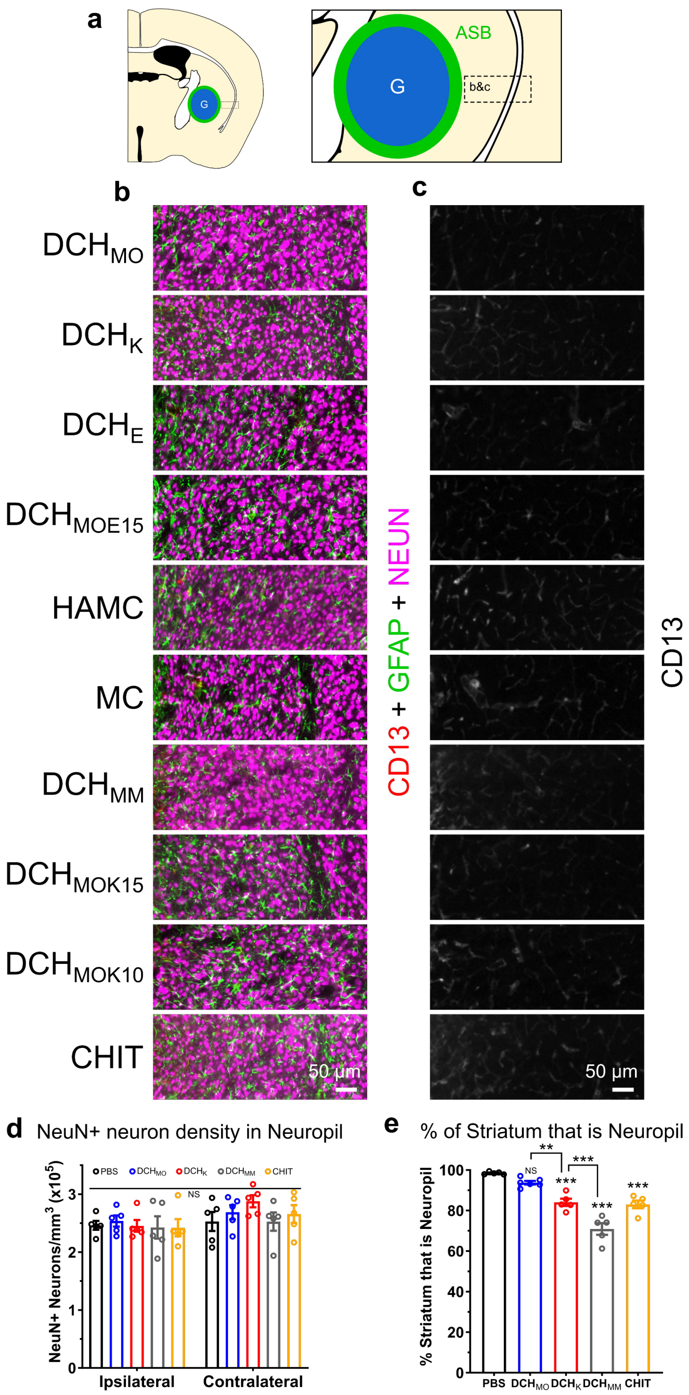

Supplementary Figure 5 | Detail analysis of neural tissue immediately adjacent to the astrocyte border (ASB) along hydrogel interfaces for the different hydrogels tested. a. Schematic of mouse forebrain indicating the location of the images in b.&c. **b.** The appearance and density of GFAP-positive astrocytes and NeuN-positive neurons in the preserved neural tissue adjacent to hydrogels was not detectably different across all samples regardless of the differences in severity of FBR in the nearby non-neural tissue. **c.** CD13-positive cells in the preserved neural tissue (thresholding of staining intensity kept constant as in FBR evaluations shown in Figure 2) were similar in appearance to those in uninjured tissue and were limited to normally appearing perivascular cells across all hydrogels tested regardless of the severity of the FBR in the nearby non-neural tissue. CD13 intensity of the perivascular cells in preserved neural tissue is considerably lower compared to the non-neural FBR across all samples. **d.** Quantification of NeuN-positive neurons in preserved neuropil for different hydrogels (DCH$_{MO}$, DCH$_{K}$, DCH$_{MM}$ and CHIT) showed no significant (NS) change in the density of neurons in the preserved neuropil despite differences in FBR severity, two-way ANOVA with Bonferroni. **e.** The percentage of striatal tissue volume at the center of the hydrogel injection that was neuropil decreased significantly with increasing severity of FBR. ***$P<0.0001$ versus PBS, **$P=0.0058$ DCH$_{MO}$ versus DCH$_{K}$ and ***$P=0.0003$ DCH$_{MM}$ versus DCH$_{K}$, one-way ANOVA with Bonferroni. All graphs show mean ± s.e.m with n=5 mice per group.

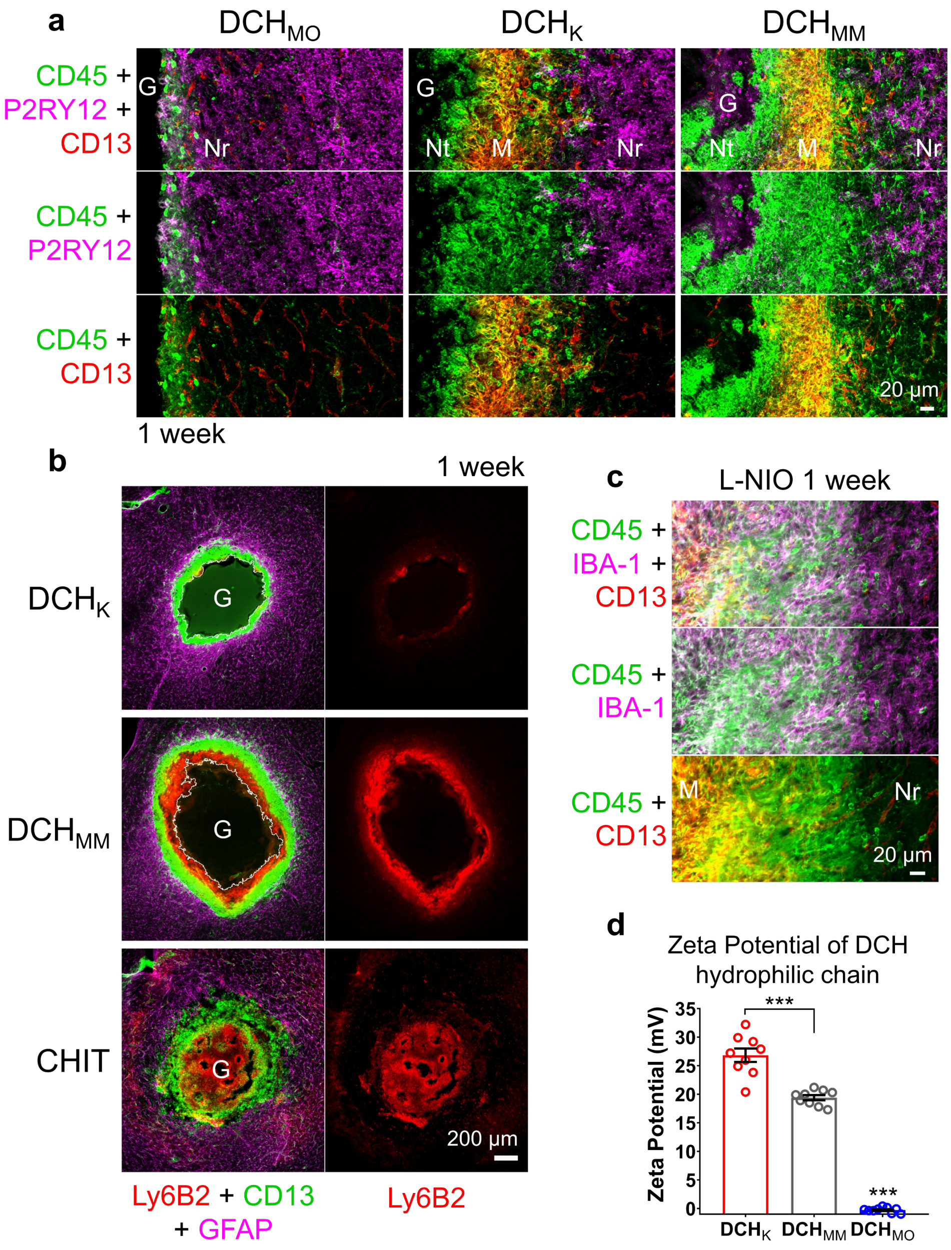

Supplementary Figure 6 | Characterization of inflammatory cells involved in hydrogel FBR or CNS wound response. a. Detail images of the escalating recruitment of peripherally derived inflammatory cells by DCH_{MO}, DCH_{K}, DCH_{MM} at one week after injection. For all hydrogels (G), the neural tissue (Nr) immediately adjacent to deposits contains reactive, CNS-derived microglia that are Iba-1-positive, P2RY12-positive and CD13-negative. Between neural tissue and hydrogels there are zones of infiltrating macrophages (M) that are CD13-positive and CD45-positive and P2RY12-negative and that increase in thickness and intensity from DCH_{MO} to DCH_{K} and DCH_{MM}. Between the layer of macrophages and DCH_{K} and DCH_{MM} there are increasing levels of CD45-positive, CD13-negative and P2YR12-negative cells that stain for Ly6B2 (see panel b) and are neutrophils (Nt). **b.** Survey images of different cationic hydrogels comparing infiltration Ly6B2-postive neutrophils at one week after injection. Note for DCH_{K}, the sparse number of neutrophils (red) between the layer CD-13 positive macrophages (green) and gel deposit, in contrast with the thick layer of neutrophils in DCH_{MM} and the robust neutrophil invasion of the entire center of the chitosan (CHIT) deposit. **c.** Detail images of an L-NIO induced stroke lesion at one week after injection. The recruitment of inflammatory cells to a stroke lesion occurs in a similar manner to that seen for hydrogel with formation of a non-neural lesion core of predominantly stromal cells and macrophages surrounded by surviving neural tissue. **d.** Zeta Potential measurements for the hydrophilic chain of DCH showing that DCH_{K} has a greater overall cationic charge compared to DCH_{MM} because the latter is a statistical copolymer that includes 10% uncharged alanine. DCH_{MO} has essentially zero charge. ***$P<0.0001$ versus DCH_{K}, one-way ANOVA with Bonferroni. Graph shows mean ± s.e.m with individual data points superimposed showing n=9 independent samples per group.

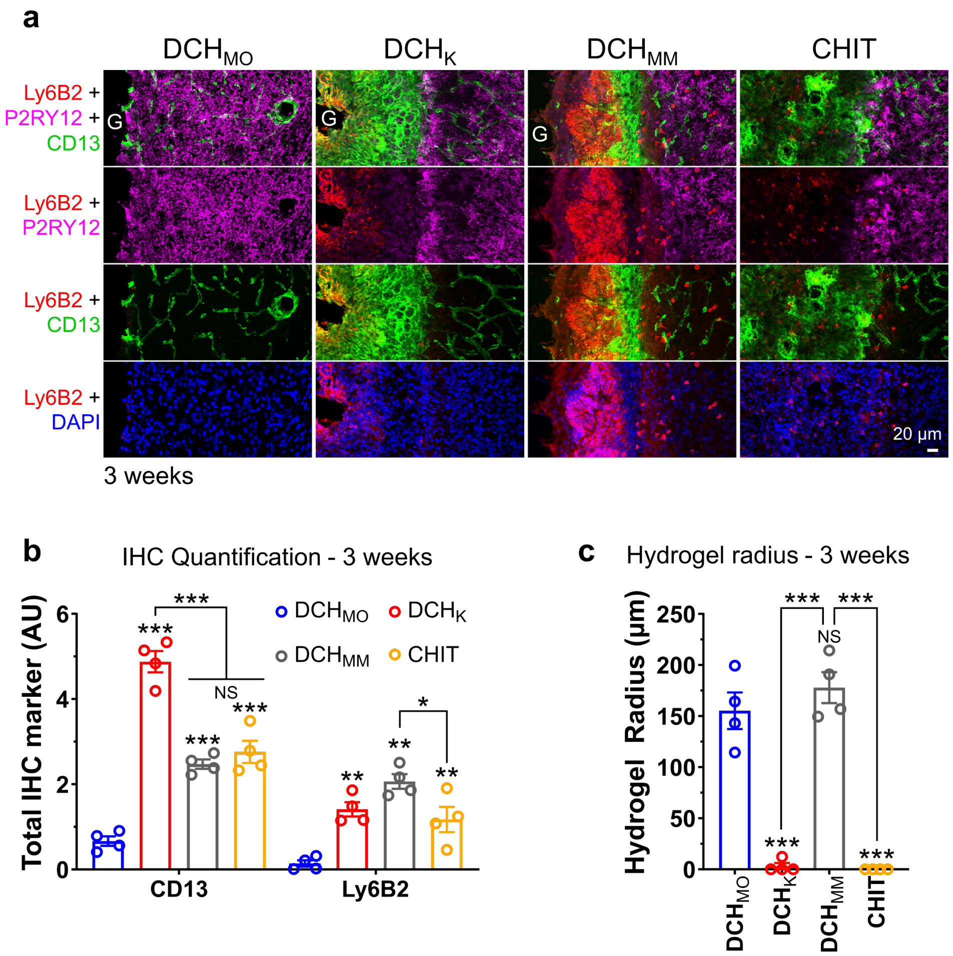

Supplementary Figure 7 | Characterization of inflammatory cells involved in hydrogel FBR at 3 weeks after injection. a. Detail images of hydrogel-tissue interface showing the distribution of neutrophils (Ly6B2), macrophages (CD13) and microglia (P2RY12) at 3 weeks for different hydrogels. Persistence of Ly6B2-positive neutrophils at the cationic DCH_{MM} hydrogel surface at three weeks after injection is apparent. DCH_{K} and chitosan (CHIT) hydrogels show infiltration of macrophages into hydrogels at 3 weeks. **b.** Quantification of total CD13 and Ly6B2 staining for hydrogels at three weeks after injection. ***P<0.0001 for all hydrogels versus DCH_{MO} for CD13; **P=0.0007, <0.0001, and =0.0063 for DCH_{K}, DCH_{MM} and CHIT versus DCH_{MO} respectively for Ly6B2, ***P<0.0001 for DCH_{MM} and CHIT versus DCH_{K} for CD13, Not Significant (NS) and *P=0.0217 for DCH_{MM} versus CHIT for CD13 and Ly6B2 respectively, two-way ANOVA with Bonferroni. **c.** Quantification of hydrogel radius for the different materials at 3 weeks. DCH_{K} and CHIT are mostly resorbed at the 3-week time point. DCH_{MM} is not consumed despite prevalent inflammatory cells at the hydrogel interface indicating frustrated phagocytosis. DCH_{MO} is also not resorbed but shows limited peripheral inflammatory cells at the hydrogel interface. NS and ***P<0.0001 for all hydrogels versus DCH_{MO} and ***P<0.0001 for DCH_{K} and CHIT versus DCH_{MM}, one-way ANOVA with Bonferroni. All graphs show mean ± s.e.m with individual data points superimposed showing n=4 mice per group.

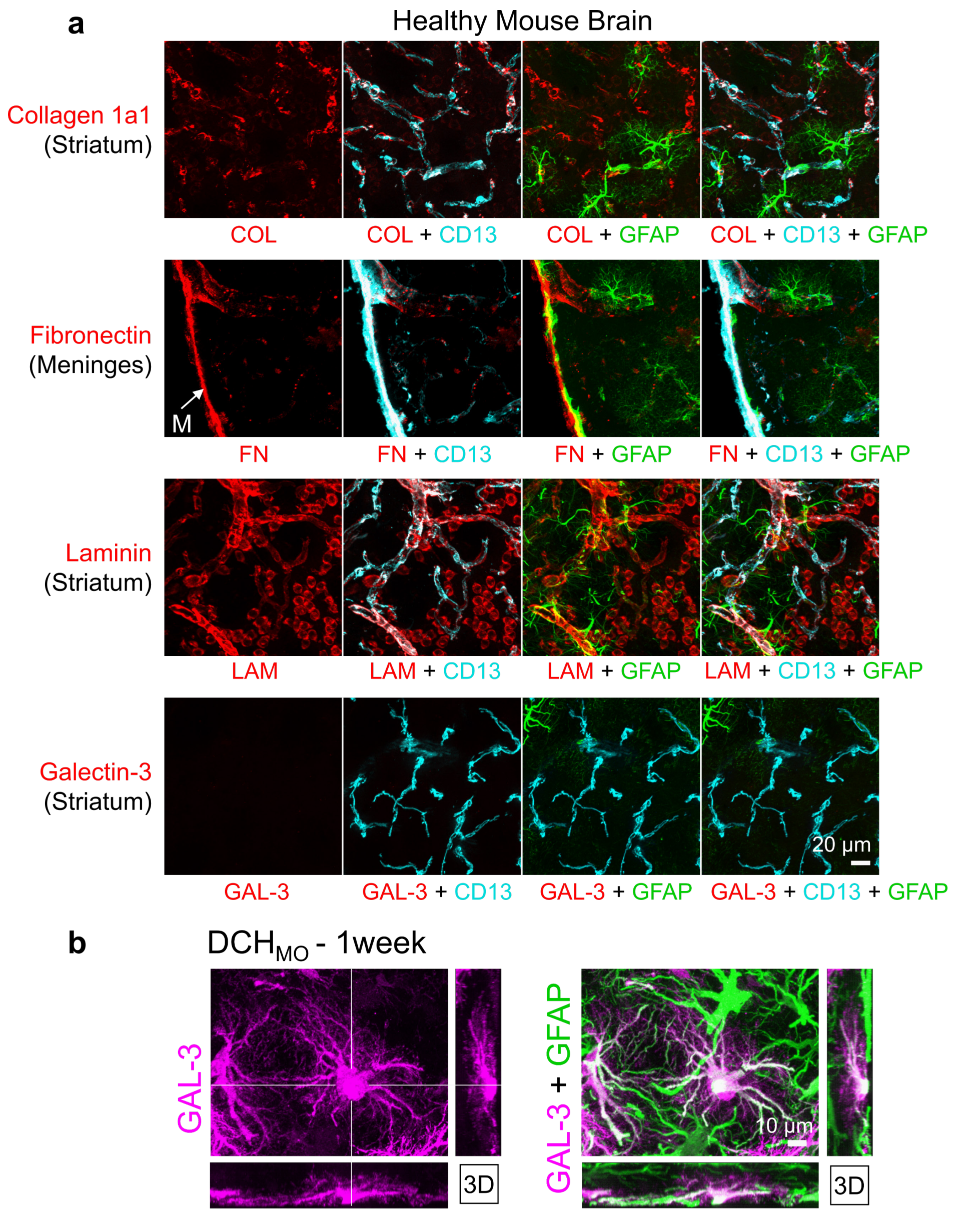

Supplementary Figure 8 |Characterization of fibrosis and stromal cell elements in healthy and hydrogel injected CNS. a. Detail images of normal wildtype mouse brain stained for extracellular matrix and stromal cell associated markers collagen 1a1, fibronectin, laminin and galectin-3. CD13-positive cells along blood vessels are also positive for collagen-1a1, fibronectin and laminin positive. CD13-positive cells along meninges (M) are also positive for fibronectin. Galectin-3 is undetectable in normal tissue. **b.** High magnification 3D view shows that galectin-3 is robustly expressed by some but not all reactive astrocytes near the DCH$_{MO}$ interface at one week. GFAP and galectin-3 colocalization is seen as white staining. Galectin-negative astrocytes are stained only green.

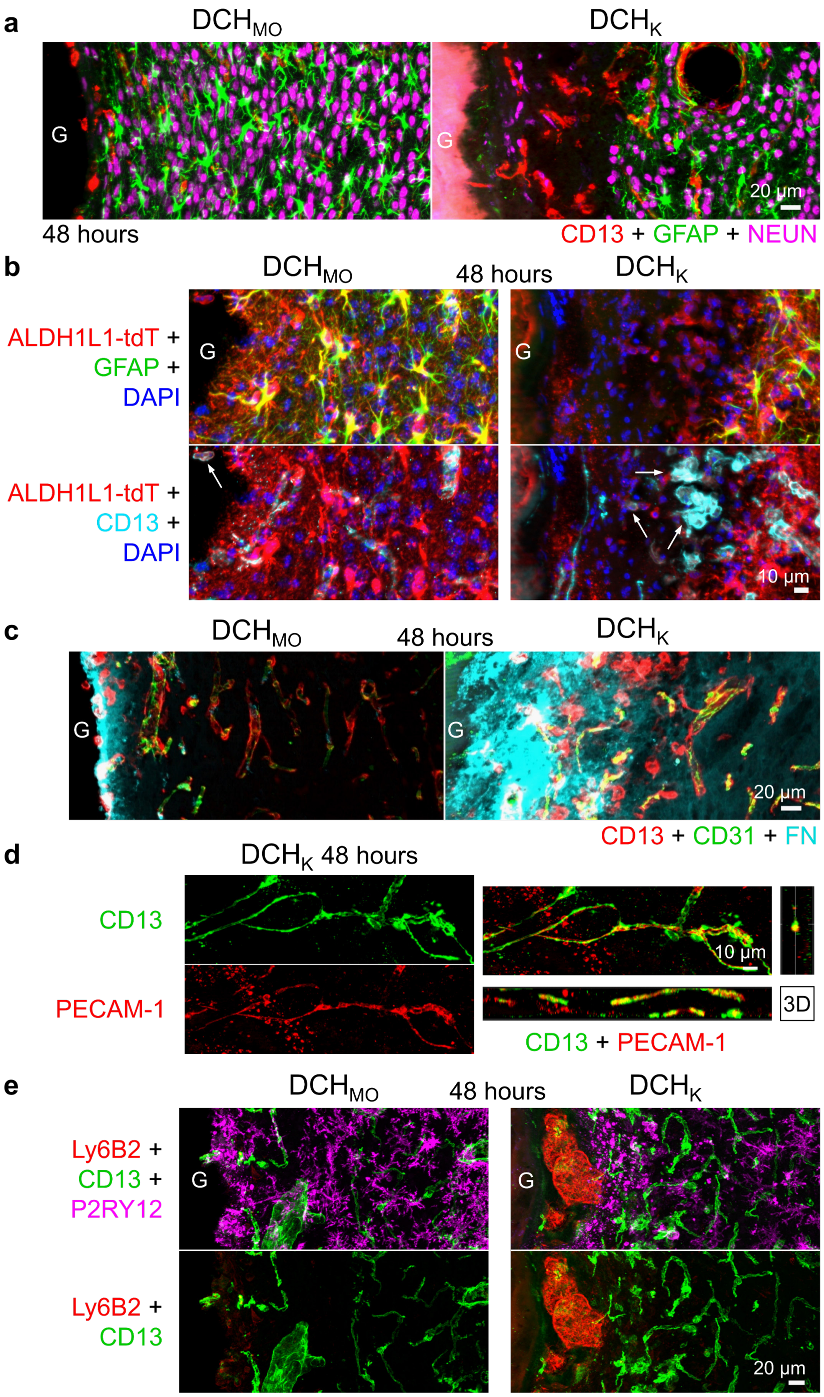

Supplementary Figure 9 | Characterization of hydrogel evoked tissue damage. a. Comparison of the tissue interface for DCH_{MO} and DCH_K at 48 hours. Note the clear zone of neural tissue loss in DCH_K indicated by loss of GFAP and NeuN positive cells and presence of CD13 cells, whereas such tissue loss is mostly absent for DCH_{MO}. **b.** Hydrogel induced loss of astrocytes in Aldh1l1-tdT mice results in tdT reporter debris within the damaged tissue zone that is phagocytosed by CD13-positive macrophages (white arrows). **c.** Neural tissue damage at the hydrogel interface results in extracellular deposition of extravasated blood-borne fibronectin, which is more apparent in DCH_K compared to DCH_{MO}. **d.** CD13 and PECAM-1 positive thin vessels in the zone of tissue damage at the DCH_K tissue interface. **e.** Ly6B2 positive neutrophils infiltrate the region of tissue damage that occurs at the interface of host tissue with DCH_K, but not at the interface with DCH_{MO}.

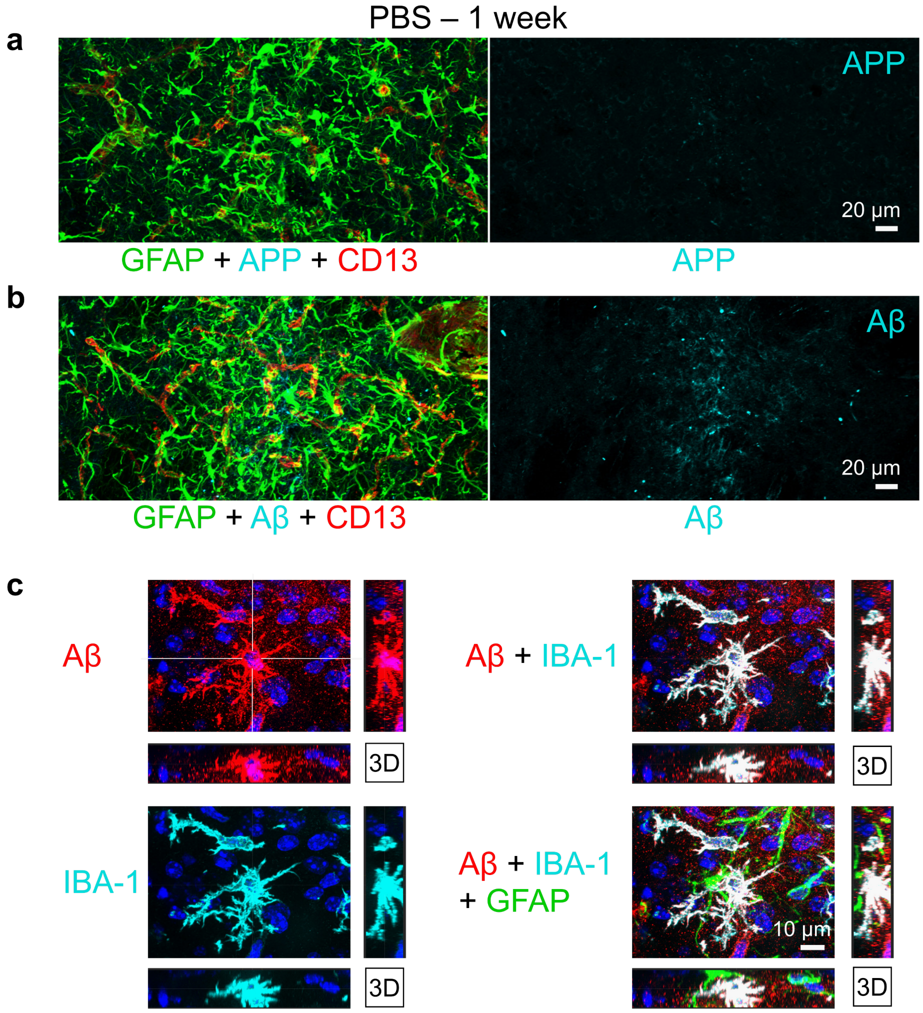

Supplementary Figure 10 | Characterization of APP and Aβ after injection of PBS or hydrogel. a,b. Infusions of PBS cause limited axonal damage, APP accumulation (**a**) and Aβ formation (**b**) only in narrow zone of damage caused by the needle track. **c.** After injection of DCH_K, Aβ is colocalized prominently within IBA-1-positive microglia but does not colocalize with GFAP-positive astrocytes.

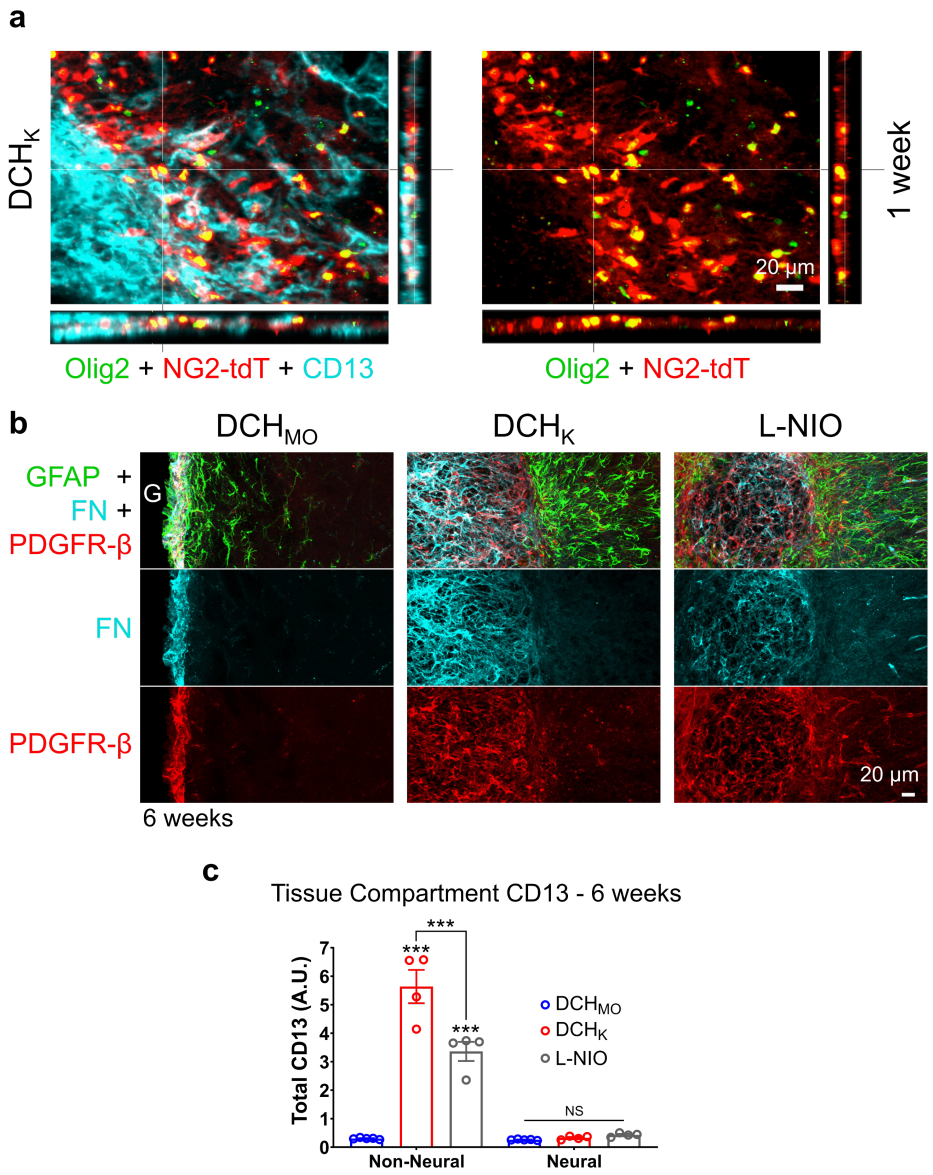

Supplementary Figure 11 | Characterization of glia and non-neural cells in chronic FBR to hydrogels. a. Detail images showing that OPCs positive for Olig2 and NG2-targeted reporter protein (NG2-tdT) remain partitioned in the neural tissue compartment during the FBR to hydrogels. **b.** Detail images showing fibronectin (FN) and PDGFR-β in the chronic fibrotic tissue abutting the mature GFAP-positive astrocyte limitans border at 6 weeks after DCH_{MO}, DCH_K and L-NIO stroke. **c.** Quantification of total CD13 staining in non-neural and neural tissue compartments for DCH_{MO}, DCH_K and L-NIO stroke after 6 weeks ***$P<0.0001$ versus DCH_{MO} and NS for neural compartment for all samples, two-way ANOVA with Bonferroni. Graph shows mean ± s.e.m with individual data points superimposed showing n=5, 4 and 4 mice for DCH_{MO}, DCH_K, and L-NIO respectively.

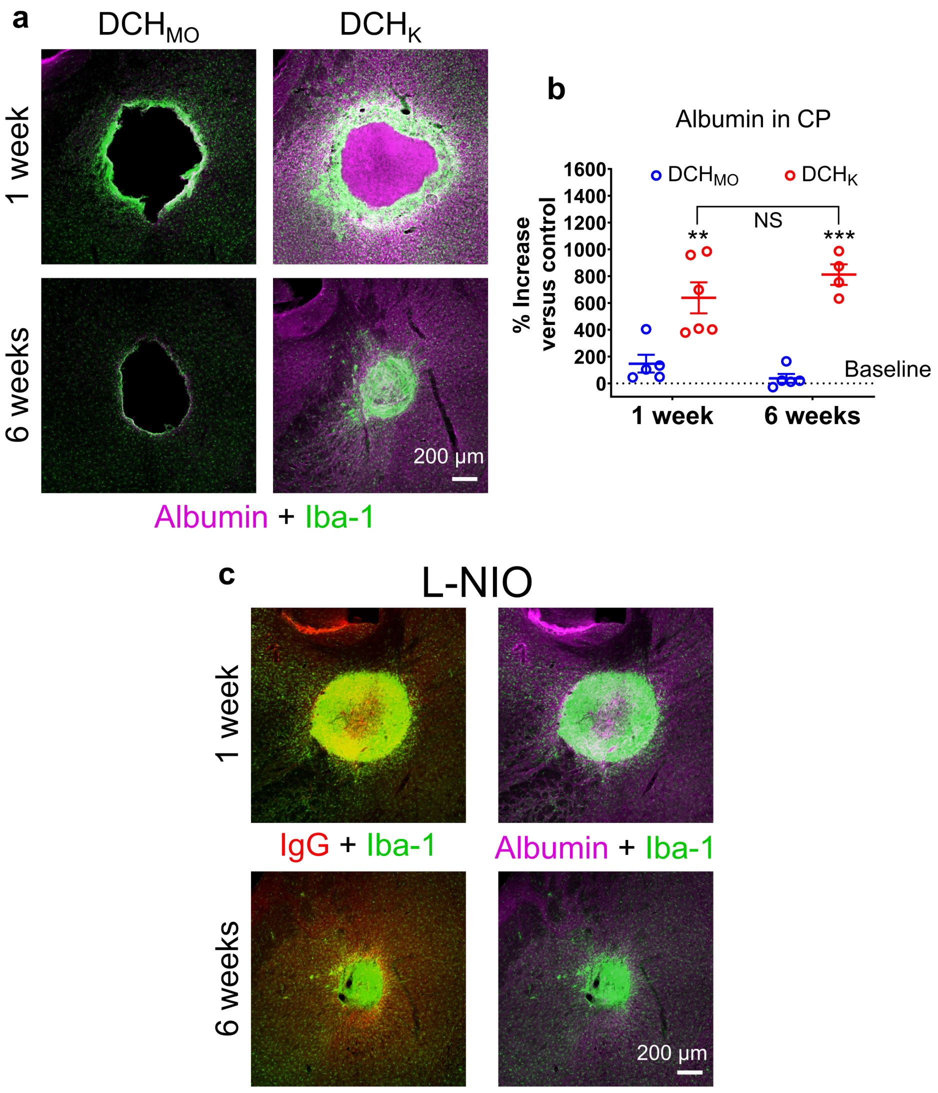

Supplementary Figure 12 | Analysis of blood-brain barrier (BBB) leak and repair following hydrogel infusions or L-NIO-induced stroke and formation of the astrocyte limitans border. a. Survey images of albumin penetration into neural parenchyma at one week and six weeks after DCH_{MO} and DCH_K injections. **b.** Quantification of the percentage increase in albumin levels in the hydrogel injected caudate putamen (CP) normalized to the non-injected contralateral side. **P=0.0012 and ***P<0.0001 for DCH_K versus DCH_{MO} at 1 week and 6 weeks respectively and NS for DCH_K samples between the two time points, two-way ANOVA with Bonferroni. Graph shows mean ± s.e.m with individual data points superimposed showing n=5 mice for DCH_{MO} at both one and six weeks and n=6 and 4 mice for DCH_K at one and six weeks respectively. **c.** Survey images of penetration serum proteins albumin and IgG into neural parenchyma at one and six weeks after L-NIO-induced strokes.

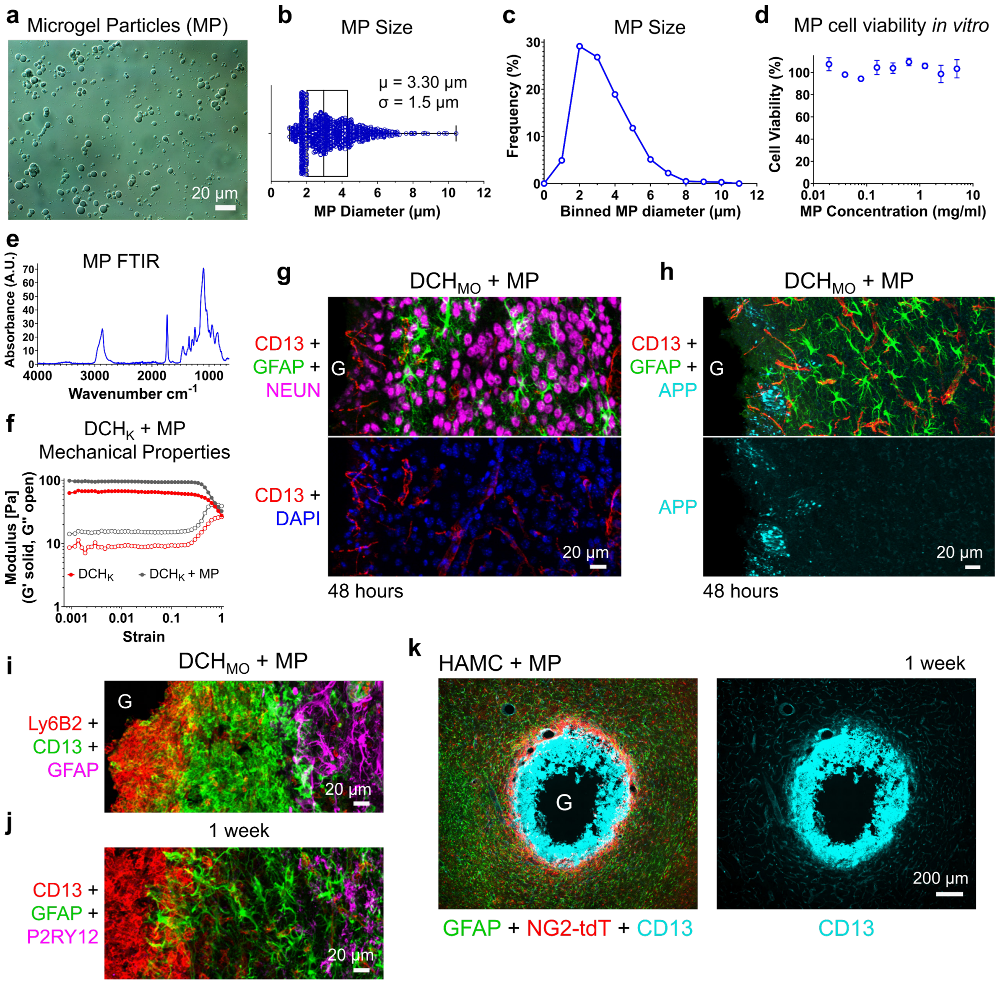

Supplementary Figure 13 | Characterization of PEG microgel particles (MP) and their effect on the FBR when combined with physical hydrogels. a. Differential interference contrast (DIC) microscopy image of PEG microgel particles (MP) formed by inverse emulsion. **b,c.** Quantification of MP diameter spread and frequency distribution showing mean(μ) and standard deviation (σ). **d.** Cell viability of cultured neural progenitor cells exposed to MP for twenty-four hours at various concentrations up to 5 mg/ml. MP are non-toxic to neural progenitors in culture at all concentrations tested (graph shows mean ± s.e.m for n=3 individual samples per concentration) **e.** ATF-FTIR spectrum of dried MP. The ester stretch (≈1730 cm^{-1}) and the loss of the acrylate C=C stretch (1675–1600 cm^{-1}) were used to confirm presence of the covalent crosslinked network. **f.** Characterization of mechanical properties of DCH loaded with MP shows that MPs impart a slight stiffening but no effect on the injectability of the hydrogel at high strain. **g,h.** Detail images showing that inclusion of MP into DCH_{MO} hydrogels (G) has no detectable adverse effects on neural tissue (g) and does not detectably increase axonal injury as identified by APP accumulation (h). **i.** Detail image showing a pronounced infiltration of CD13-positive macrophages and Ly6B2-positive neutrophils at 1 week within deposits of DCH_{MO} hydrogels loaded with MP. **j.** Detail image showing that P2Y12R-positive microglia do not infiltrate into DCH_{MO}+MP deposits. **k.** Detail image showing that loading MP into HAMC hydrogels also leads to a pronounced recruitment and infiltration of CD13-positve cells by 1 week and that GFAP-positive astrocytes and NG2-tdT-positive OPCs do not migrate into CD13 positive cell zones.

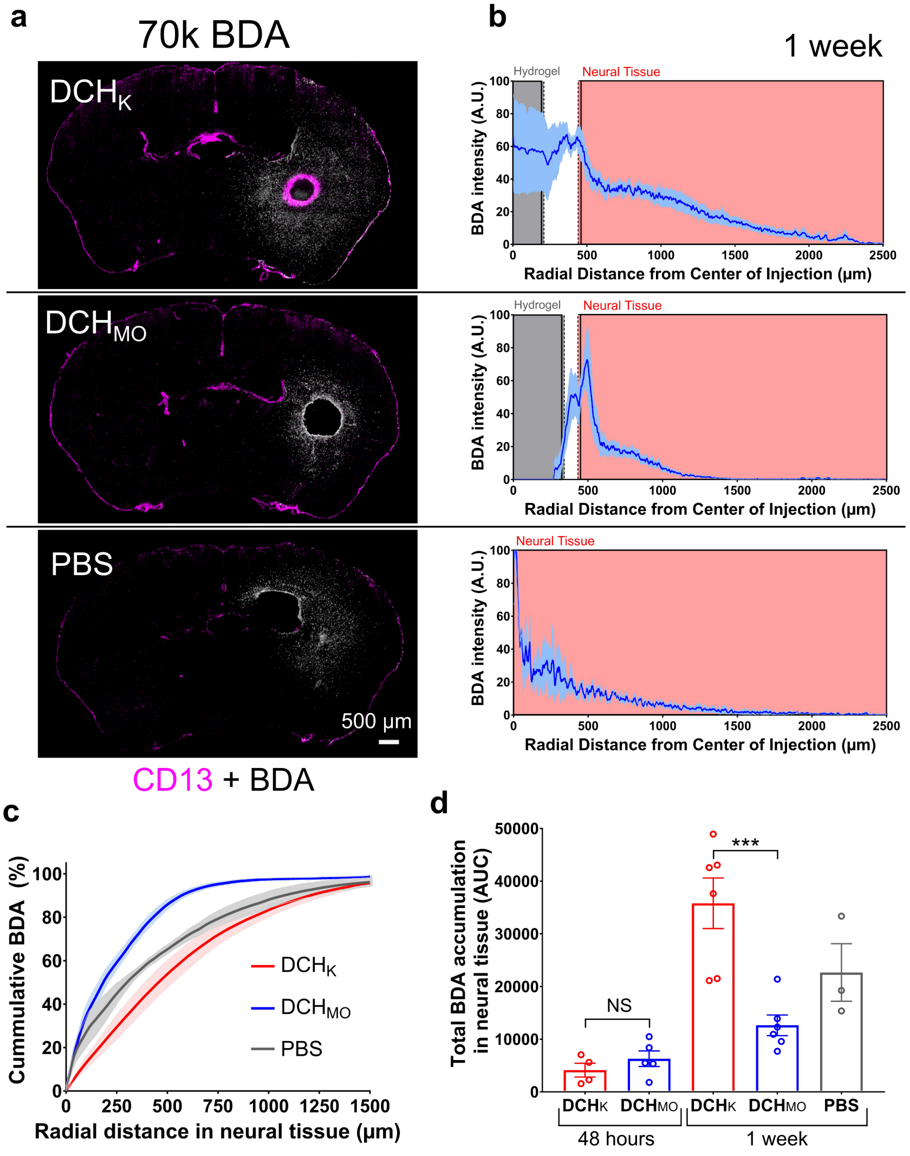

Supplementary Figure 14 | Analysis of biodistribution of 70kDA BDA (BDA-70) released from hydrogels in the striatum. a. Survey images of BDA-70 delivery using DCH_K, DCH_{MO}, and PBS carriers at 1 week. **b.** BDA intensity plots of BDA-70 released into the lateral striatum and cortex as measured radially from the center of the hydrogel injection. Grey shaded regions indicate location of the hydrogel, red shaded regions indicate neural parenchyma tissue, the white unshaded zone between the grey and red shaded regions includes non-neural tissue and start of the astrocyte limitans border. Data are mean ± s.e.m, with s.e.m. represented as light blue shaded areas around the average intensity plot for each condition. **c.** Cumulative BDA-70 accumulation in neural tissue at 1 week for the hydrogel and PBS carriers. In b. and c. data are mean ± s.e.m, with s.e.m. represented as the lighter shaded area with n= 6, 6, 3 mice for DCH_{MO}, DCH_K and PBS respectively. **d.** Total BDA-70 accumulation for the different conditions at 48 hours and 1 week. There was no difference in burst release from the two hydrogels but DCH_K showed increased accumulation of BDA-70 in neural tissue compared to DCH_{MO} at 1 week. Not significant (NS) and ***P<0.0001 for DCH_K versus DCH_{MO}, two-way ANOVA with Bonferroni. Data are mean ± s.e.m with individual data points superimposed showing *n=4, 5, 6, 6, 3* mice for DCH_K at 48 hours, DCH_{MO} at 48 hours, DCH_K at 1 week, DCH_{MO} at 1 week, and PBS at 1 week respectively.

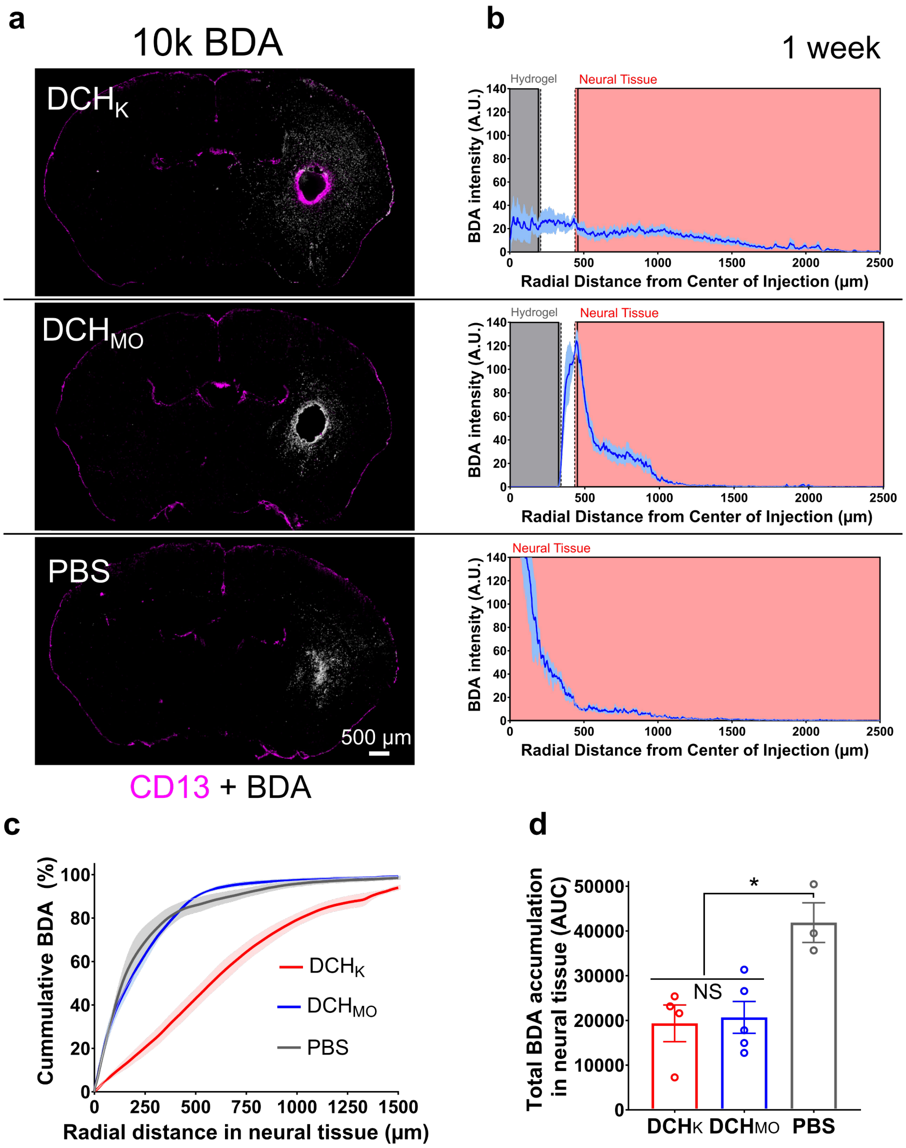

Supplementary Figure 15 | Analysis of biodistribution of 10kDA BDA (BDA-10) released from hydrogels in the striatum. a. Survey images of BDA-10 delivery using DCH$_K$, DCH$_{MO}$, and PBS carriers at 1 week. **b.** BDA intensity plots of BDA-10 released into the lateral striatum and cortex as measured radially from the center of the hydrogel injection. Data are mean ± s.e.m, with s.e.m. represented as light blue shaded areas around the average intensity plot for each condition**. c.** Cumulative BDA-10 accumulation in neural tissue at 1 week measured radially for the hydrogel and PBS carriers. DCH$_K$ shows significantly greater depth of BDA-10 penetration into neural parenchyma compared to DCH$_{MO}$. **d.** Total BDA-10 accumulation for the different conditions at 1 week shows no difference between the hydrogels but a small increased accumulation for the PBS carrier. *P=0.0163 and =0.0149 for PBS versus DCH$_{MO}$ and DCH$_K$ respectively, one-way ANOVA with Bonferroni. Data in all graphs are mean ± s.e.m with s.e.m. represented as lightly shaded banded areas in b. and c. and error bars in d. Individual data points are superimposed on bars in d. For all graphs, n=4, 5, 3 mice for DCH$_K$, DCH$_{MO}$, and PBS respectively.

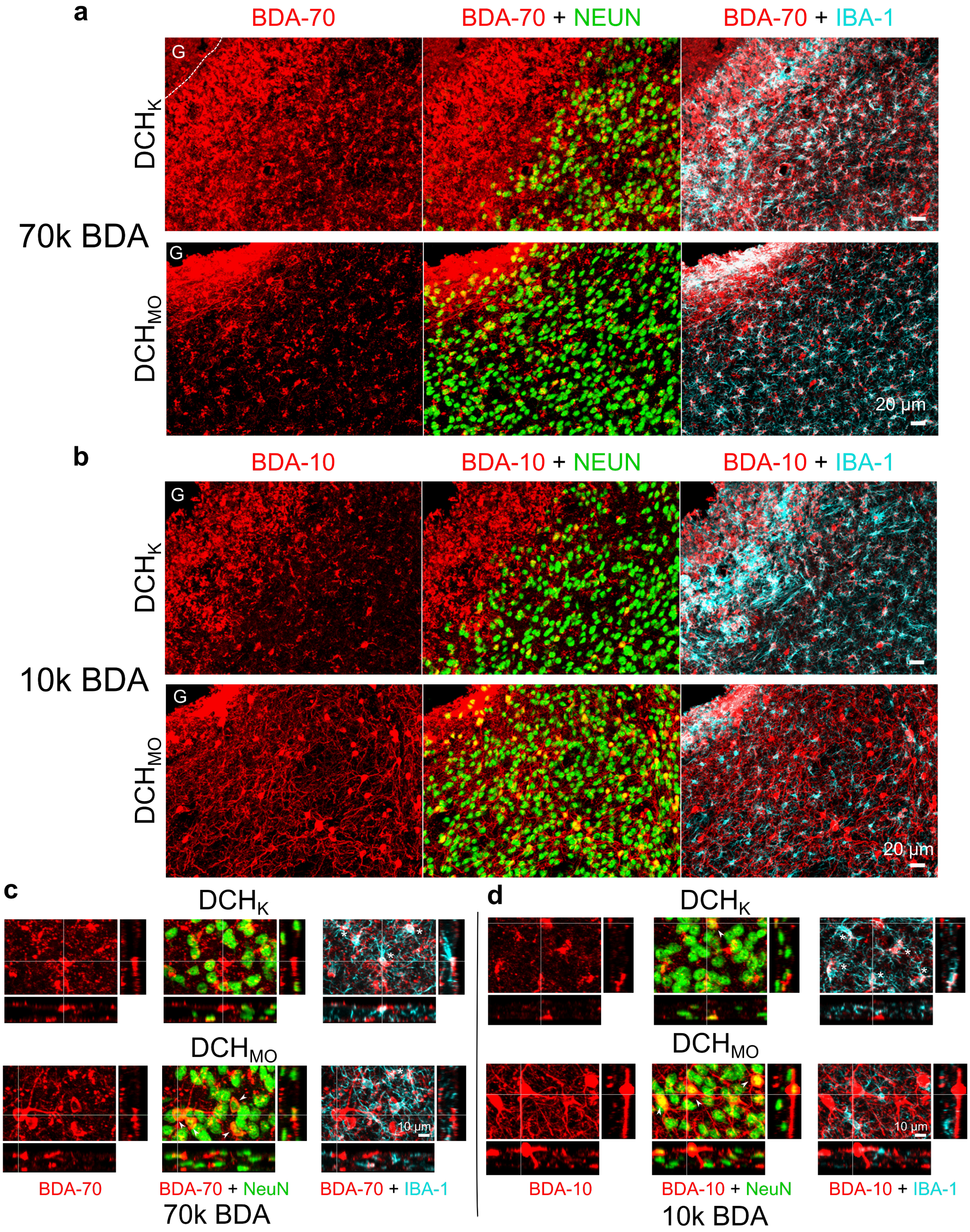

Supplementary Figure 16 | BDA delivered from hydrogels is accumulated in neurons, microglia and macrophages in a size and hydrogel dependent manner. a. Detail images showing the relative uptake of released BDA-70 by NeuN-positive neurons and IBA-1 positive microglia/macrophages at 1 week for DCH_K and DCH_{MO}. **b.** Detail images showing the relative uptake of released BDA-10 by NeuN-positive neurons and IBA-1 positive microglia/macrophages at 1 week for DCH_K and DCH_{MO}. **c. & d.** Representative Orthogonal (3D) images of BDA-70 (c.) and BDA-10 (d.) uptake in NeuN-positive neurons and IBA-1 positive microglia in the preserved striatal neuropil at 1 week for DCH_K and DCH_{MO}. Asterisk (*) markers indicate uptake of BDA by microglia and arrows indicate uptake of BDA by neurons.

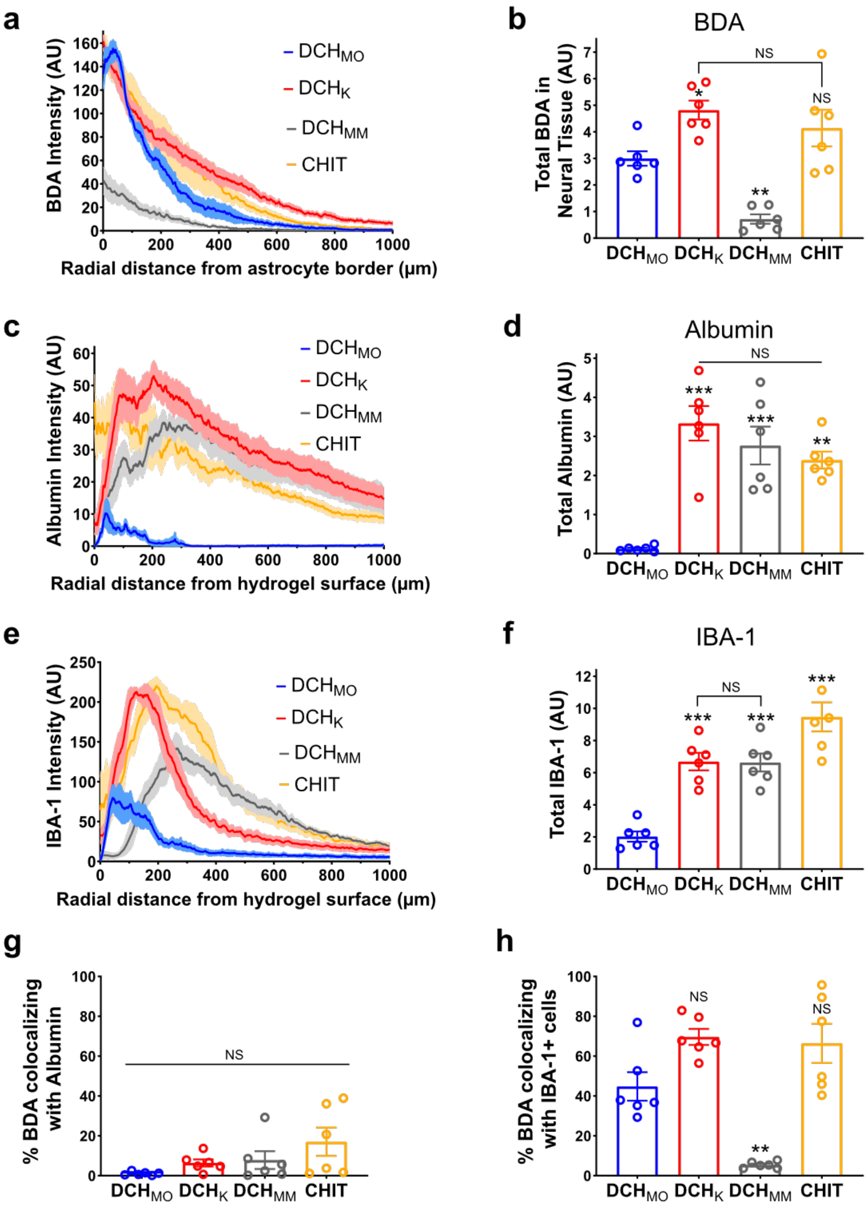

Supplementary Figure 17 | Quantification of BDA delivery and FBR for hydrogels at 2 weeks. a. BDA intensity distribution within neural parenchyma for the different hydrogels. **b.** Total BDA accumulation in neural tissue for the different hydrogel carriers. Not Significant (NS), *P=0.0364, **P=0.0063 for hydrogels versus DCH_{MO}. **c.** Average albumin intensity profile for the different hydrogels. **d.** Total albumin staining around hydrogel deposits. ***P<0.0001 and =0.0002 for DCH_{K} and DCH_{MM} respectively versus DCH_{MO}, **P=0.0009 for CHIT versus DCH_{MO}, NS for comparisons between DCH_{K}, DCH_{MM}, and CHIT. **e.** Average intensity plot for Iba-1 IHC staining measured radially from the hydrogel surface **f.** Total Iba-1 around hydrogel deposits. ***P=0.0002 (DCH_{K}), =0.0002 (DCH_{MM}) and <0.0001 (CHIT) versus DCH_{MO}. **g.** Percentage of total BDA colocalizing with albumin for the different hydrogels. There was no significant colocalization of BDA-10 with albumin for all samples **h.** Percentage of total BDA-10 colocalizing with Iba-1 for the different hydrogels. **P=0.0019 for DCH_{MM} versus DCH_{MO} and NS for DCH_{K} or CHIT versus DCH_{MO}. All statistical analyses are one-way ANOVA with Bonferroni. In a., c. and e. figures the data are mean ± s.e.m (lighter shaded banded area) for n=6 mice per group. Data in figures b., d. and f-h. are mean ± s.e.m (error bars) with individual data points superimposed showing n=6 mice per group.

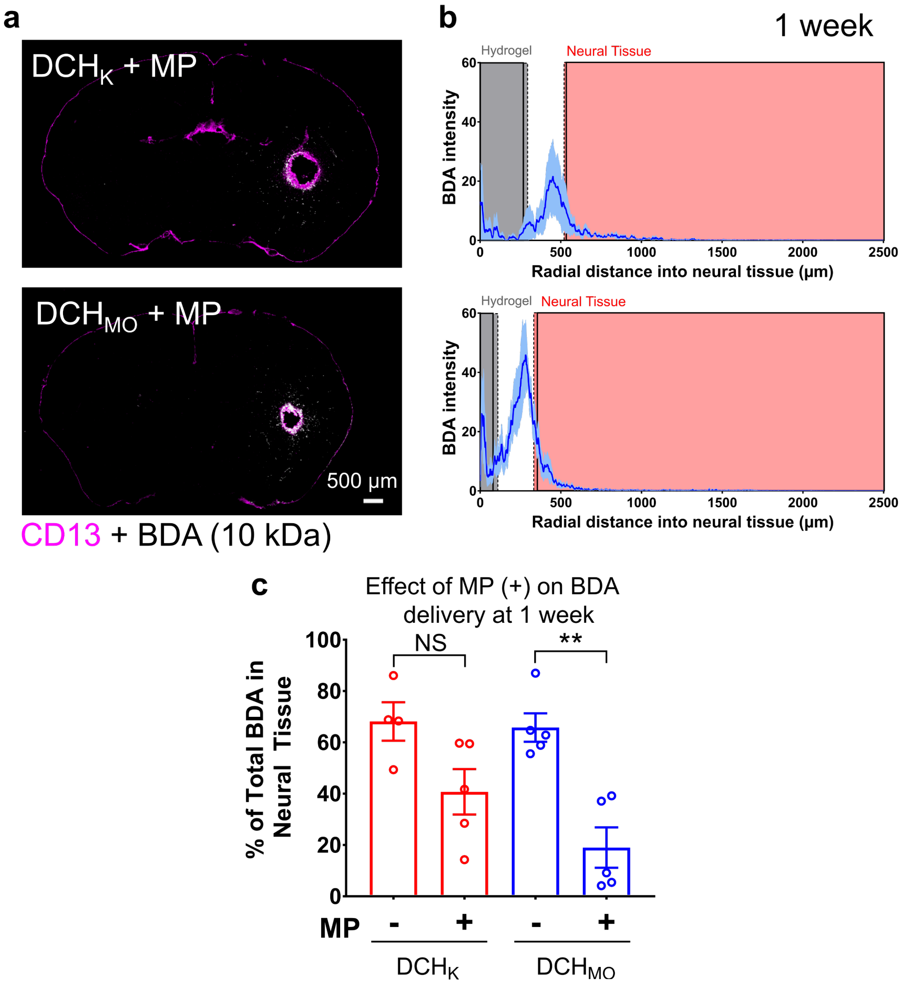

Supplementary Figure 18 | BDA delivered from microgel particles (MP) suspended in hydrogels is affected by MP dependent FBR. a. Survey images of BDA-10 biodistribution at 1 week delivered from MP that were suspended in DCH_K and DCH_{MO}. **b.** Intensity plots of BDA-10 released from MP loaded hydrogels. Grey shaded regions indicate location of the hydrogel, red shaded regions indicate neural parenchyma tissue. Data are mean ± s.e.m, with s.e.m. represented as light blue shaded areas around the average intensity plot for each condition with n=5 mice per group. **c.** Comparisons of the Percentage of total BDA accumulated in neural tissue at 1 week for DCH_K and DCH_{MO} when delivered with (+) or without (-) MP. For DCH_{MO} there was a significant decrease in the amount of BDA-10 delivered to neural parenchyma with the use of the MP, **P=0.0026 for DCH_{MO}+MP versus DCH_{MO} only, one-way ANOVA with Bonferroni. Data are mean ± s.e.m with individual data points showing n=4, 5, 5, 5 mice for DCH_K, DCH_K+MP, DCH_{MO}, and DCH_{MO}+MP respectively.

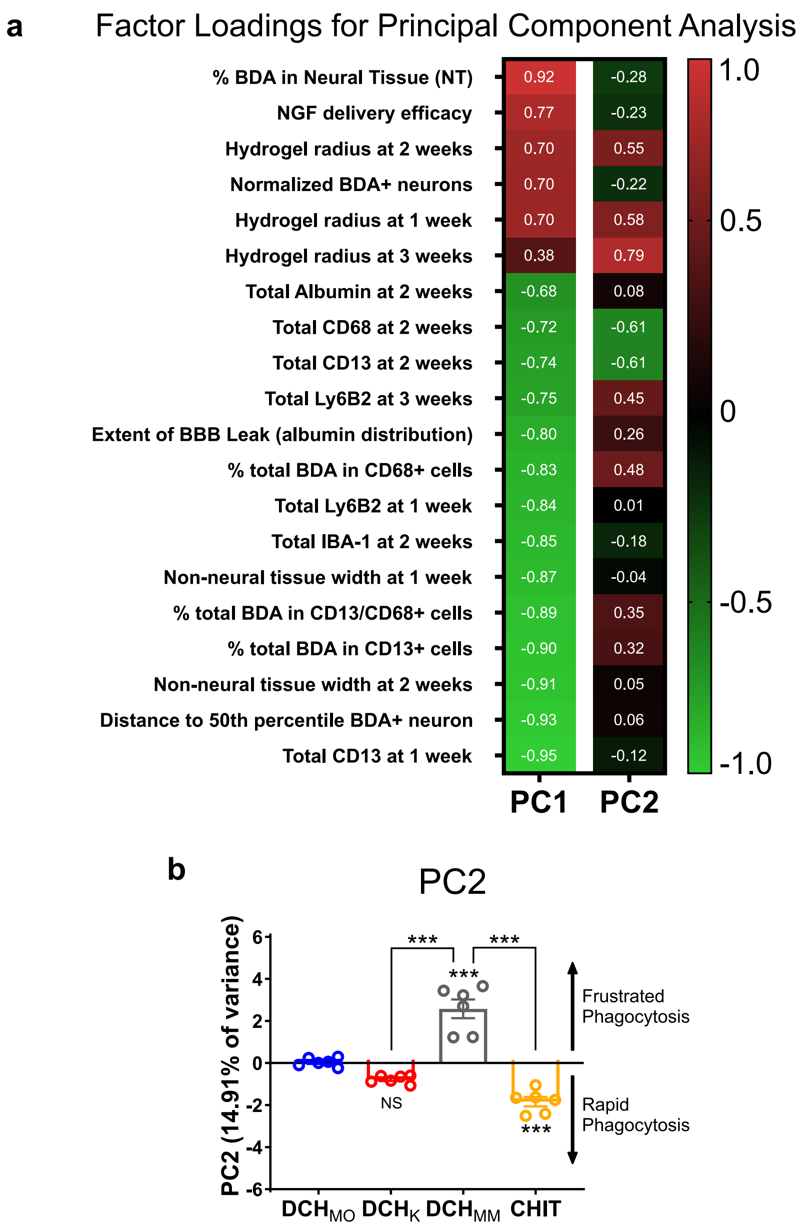

Supplementary Figure 19 | Principal Component Analysis (PCA) to assess hydrogel function in the CNS. a. List of parameters used in the PCA with their corresponding factor loadings for the orthogonal PC1 and PC2 axes. The parameters included in the PCA had a Kaiser-Meyer-Olkin measure of sampling adequacy (KMO) of greater than 0.5. **b.** Graphical representation of the positions of the different hydrogel samples along the PC2 axis which accounts for 14.91% of the total variance. NS for DCH_K, ***P<0.0001 for DCH_{MM} and =0.0003 for CHIT versus DCH_{MO} respectively and ***P < 0.0001 for DCH_K or CHIT versus DCH_{MM}. Data are mean ± s.e.m with individual data points superimposed showing n=6 mice per group.

Supplementary References

[1] Wollenberg, A.L., et al., *Injectable polypeptide hydrogels via methionine modification for neural stem cell delivery.* Biomaterials, 2018. **178**: p. 527-545.

[2] Nowak, A.P., et al., *Rapidly recovering hydrogel scaffolds from self-assembling diblock copolypeptide amphiphiles.* Nature, 2002. **417**(6887): p. 424-8.

[3] Yang, C.-Y., et al., *Biocompatibility of amphiphilic diblock copolypeptide hydrogels in the central nervous system.* Biomaterials, 2009. **30**(15): p. 2881-2898.

[4] Song, B., et al., *Sustained local delivery of bioactive nerve growth factor in the central nervous system via tunable diblock copolypeptide hydrogel depots.* Biomaterials, 2012. **33**(35): p. 9105-16.
