# Supplementary material for: Foreign body responses in mouse central nervous system mimic natural wound responses and alter biomaterial functions
Source: Nat Commun. 2020 Dec 4;11:6203. doi: 10.1038/s41467-020-19906-3 (PMC7718896; doi:10.1038/s41467-020-19906-3)
Supplement: Supplementary file 2 — Reporting Summary [file 41467_2020_19906_MOESM2_ESM.pdf]

nature research

Corresponding author(s): Michael V Sofroniew

Last updated by author(s): Oct 8, 2020

Reporting Summary

Nature Research wishes to improve the reproducibility of the work that we publish. This form provides structure for consistency and transparency in reporting. For further information on Nature Research policies, see our Editorial Policies and the Editorial Policy Checklist.

Statistics

For all statistical analyses, confirm that the following items are present in the figure legend, table legend, main text, or Methods section.

n/a	Confirmed	
☐	☒	The exact sample size (*n*) for each experimental group/condition, given as a discrete number and unit of measurement
☐	☒	A statement on whether measurements were taken from distinct samples or whether the same sample was measured repeatedly
☐	☒	The statistical test(s) used AND whether they are one- or two-sided<br>*Only common tests should be described solely by name; describe more complex techniques in the Methods section.*
☒	☐	A description of all covariates tested
☐	☒	A description of any assumptions or corrections, such as tests of normality and adjustment for multiple comparisons
☐	☒	A full description of the statistical parameters including central tendency (e.g. means) or other basic estimates (e.g. regression coefficient) AND variation (e.g. standard deviation) or associated estimates of uncertainty (e.g. confidence intervals)
☐	☒	For null hypothesis testing, the test statistic (e.g. *F*, *t*, *r*) with confidence intervals, effect sizes, degrees of freedom and *P* value noted<br>*Give P values as exact values whenever suitable.*
☒	☐	For Bayesian analysis, information on the choice of priors and Markov chain Monte Carlo settings
☒	☐	For hierarchical and complex designs, identification of the appropriate level for tests and full reporting of outcomes
☒	☐	Estimates of effect sizes (e.g. Cohen's *d*, Pearson's *r*), indicating how they were calculated

Our web collection on statistics for biologists contains articles on many of the points above.

Software and code

Policy information about availability of computer code

Data collection	NIH Image J (1.51), G*Power Software V 3.1.9.2., Microsoft Excel for Microsoft Office 365 ProPlus, Imaris 9.2 (Bitplane), Prism 8 (GraphPad Software Inc), and Aperio Imagescope v12.3 (Leica) were commercially available software used in data collection.
Data analysis	Microsoft Excel for Microsoft Office 365 ProPlus, Prism 8 (GraphPad Software Inc), XLStat Basic 2020.3.1 (Addinsoft Inc), and ChemDraw 18.2 were commercially available software used in data analysis.

For manuscripts utilizing custom algorithms or software that are central to the research but not yet described in published literature, software must be made available to editors and reviewers. We strongly encourage code deposition in a community repository (e.g. GitHub). See the Nature Research guidelines for submitting code & software for further information.

Data

Policy information about availability of data

All manuscripts must include a data availability statement. This statement should provide the following information, where applicable:

- Accession codes, unique identifiers, or web links for publicly available datasets
- A list of figures that have associated raw data
- A description of any restrictions on data availability

All data generated for this study are included in the main and supplementary figures. For all quantitative figures, files of source data of individual values as well as the results of statistical tests are provided with the paper. Other data that support the findings of this study are available on reasonable request from the corresponding author.

April 2020

Field-specific reporting

Please select the one below that is the best fit for your research. If you are not sure, read the appropriate sections before making your selection.

☒ Life sciences ☐ Behavioural & social sciences ☐ Ecological, evolutionary & environmental sciences

For a reference copy of the document with all sections, see nature.com/documents/nr-reporting-summary-flat.pdf

Life sciences study design

All studies must disclose on these points even when the disclosure is negative.

Sample size	For in vivo experiments the animal group sizes were calculated to provide at least 80% power when using the following parameters: probability of type I error (alpha) = .05, a conservative effect size of 0.25, 3-10 treatment groups with multiple measurements obtained per replicate. For all experiments performed as part of this paper the groups sizes are reported. For all other experiments no specific power analysis was used with samples sizes determined by following general standards of the field.
Data exclusions	No data were excluded from analysis.
Replication	In vivo experiments that involved injections of hydrogel formulations and/or stroke inducing agent L-NIO were repeated independently at least three times in different cohorts of mice across a three-year period with similar results. Key data generated from the immunohistochemistry analysis were repeated independently by two co-authors. All replications were successful.
Randomization	Animals were randomly assigned numbers and thereafter were evaluated blind to experimental condition. Across all experiments animals were randomized for weight, age and sex.
Blinding	Animals were randomly assigned numbers and thereafter were evaluated blind to experimental condition throughout immunohistochemical processing and imaging.

Reporting for specific materials, systems and methods

We require information from authors about some types of materials, experimental systems and methods used in many studies. Here, indicate whether each material, system or method listed is relevant to your study. If you are not sure if a list item applies to your research, read the appropriate section before selecting a response.

Materials & experimental systems

n/a	Involved in the study	
☐	☒	Antibodies
☒	☐	Eukaryotic cell lines
☒	☐	Palaeontology and archaeology
☐	☒	Animals and other organisms
☒	☐	Human research participants
☒	☐	Clinical data
☒	☐	Dual use research of concern

Methods

n/a	Involved in the study	
☒	☐	ChIP-seq
☒	☐	Flow cytometry
☒	☐	MRI-based neuroimaging

Antibodies

Antibodies used	The primary antibodies used in this study were: rabbit anti-GFAP (1:1000; Cat#Z-0334, Dako, Santa Clara, CA); rat anti-GFAP (1:1000, Cat#13-0300, Clone-2.2B10, Thermofisher, Grand Island, NY); rabbit anti NeuN (1:1000, Cat#Ab177487, Clone-EPR12763, Abcam, Cambridge, MA); guinea pig anti-NeuN (1:1000, Cat#266-004, Synaptic Systems, Goettingen, Germany); goat anti-CD13 (1:200, Cat#AF2335, R&D systems, Minneapolis, MN); rabbit anti-Laminin 1 (1:100, Cat#L-9393, Sigma, St.Louis, MO); rabbit anti-Fibronectin (1:500, Cat#AB2033, Millipore, Burlington, MA); rabbit anti-Collagen 1a1 (1:300, Cat#NB600-408. Novus Biologicals, Littleton, CO); rabbit anti-RFP (1:1000, Cat#600-401-379, Rockland, Limerick, PA); goat anti-Albumin (1:300, Cat#NB600-41532, Novus Biologicals, Littleton, CO); rat anti-PECAM-1 (1:200, Cat#550274, clone-MEC 13.3 BD Biosciences, San Jose, CA); rat anti-Galectin-3 (1:200, Cat#14-5301-82, Clone-M3/38, Invitrogen-Thermofisher Scientific, Grand Island, NY); rat anti-CD68 (1:1000, Cat# MCA1957, CLone-FA-11, AbDserotec-BioRad, Hercules, CA); rat anti-CD45 (1:100, Cat#553076, Clone-30-F11, BD Biosciences, San Jose, CA); rabbit anti-Iba-1 (1:800, Cat#019-19741, Wako, Osaka, Japan); guinea pig anti-Iba-1 (1:800, Cat#234-004, Synaptic systems, Goettingen, Germany); Rabbit anti-P2Y12R (1:500, Cat#AS-55043A, Anaspec, Fremont, CA); rabbit anti-mouse IgG (1:1000, Cat#97042, Abcam, Cambridge, MA); rat anti-Ly6B2 (1:200, Cat#MCA771GT, Clone-7/4, Bio-Rad, Hercules, CA); Goat anti-PDGFR-β (1:200, Cat#AF1042, R&D systems, Minneapolis, MN); and rabbit anti-Olig2 (1:200, Cat#AB9610, Millipore, Burlington, MA). All secondary antibodies used in this study were purchased from Jackson ImmunoResearch (West Grove, PA). Primary antibodies without a designated clone are polyclonal. All secondary antibodies were affinity purified whole IgG(H+L) antibodies with donkey host and target species dictated by the specific primary antibody used.
Validation	All antibodies used were sourced from commercial vendors and were selected because they had previously been validated for use on

Validation	mouse tissue (validated mouse reactivity) and for use in fluorescent immunohistochemistry (IHC) applications. Furthermore, validation of these antibodies can be found in peer reviewed publications by our team and others which are referenced throughout the manuscript. Additional validation information of each antibody is available from the various manufacturers' websites and validation information for each individual antibody is provided below.<br>Rabbit anti-GFAP (1:1000; Cat#Z-0334, Dako, Santa Clara, CA) validated and used consistently over many publications (e.g. (Anderson et al. Nature. 2016 & 2018), validated by Dako and information available on their website, used extensively across many other publications (~1839 citations on CiteAb);<br>Rat anti-GFAP (1:1000, Cat#13-0300, Thermofisher, Grand Island, NY) validated by company on their website and used within the concentration range recommended for IHC, cross-validated by us by comparing with the Rabbit GFAP above;<br>Rabbit anti NeuN (1:1000, Cat#Ab177487, Abcam, Cambridge, MA) - information describing validation of concentration and for use with mouse on company website, used at recommended concentration for IHC by manufacturer and used by us extensively in previous studies (Anderson et al. Nature 2018);<br>Guinea pig anti-NeuN (1:1000, Cat#266-004, Synaptic Systems, Goettingen, Germany) validation information on company website, and our own successful validation was made by comparing with Rabbit anti-NeuN above.<br>Goat anti-CD13 (1:200, Cat#AF2335, R&D systems, Minneapolis, MN) - validation information on company website and used by us at recommended concentration range in previous studies (e.g. Anderson et al. Nature. 2018);<br>Rabbit anti-Laminin 1 (1:100, Cat#L-9393, Sigma, St.Louis, MO) - validation information on company website and used within recommended dilution range by us extensively in previous studies (e.g. Anderson et al. Nature. 2018);.<br>rabbit anti-Fibronectin (1:500, Cat#AB2033, Millipore, Burlington, MA) - validation information on company website and used by us extensively in previous studies (e.g. Anderson et al. Nature. 2018);<br>Rabbit anti-Collagen 1a1 (1:300, Cat#NB600-408. Novus Biologicals, Littleton, CO) - validation information on company website and used by us extensively in previous studies (e.g. Anderson et al. Nature. 2018);<br>Rabbit anti-RFP (1:1000, Cat#600-401-379, Rockland, Limerick, PA) validation information on company website and confirmed by us by comparing endogenous tdT expression with RFP stained antibody sections.<br>Goat anti-Albumin (1:300, Cat#NB600-41632, Novus Biologicals, Littleton, CO)- validation information on company website and used within specified range for IHC applications.<br>Rat anti-PECAM-1 (1:200, Cat#550274, BD Biosciences, San Jose, CA)- validation information on company website and used by us before in previous publications (e.g. Anderson et al. 2018. Nature);<br>Rat anti-Galectin-3 (1:200, Cat#14-5301-82 Invitrogen-Thermofisher Scientific, Grand Island, NY)- validation information on company website, manufacturer states that "Antibody was verified by Knockdown to ensure that the antibody binds to the antigen stated" and used at the specified concentration for IHC provided by the manufacturers;<br>Rat anti-CD68 (1:1000, Cat# MCA1957, AbDserotec-BioRad, Hercules, CA)-validation information on company website and used previously by us in publications;<br>Rat anti-CD45 (1:100, Cat#553076, BD Biosciences, San Jose, CA)- validation information on company website and used previously by us in publications;<br>Rabbit anti-Iba-1 (1:800, Cat#019-19741, Wako, Osaka, Japan)-validation information on company website and used previously by us in publications;<br>Guinea pig anti-Iba-1 (1:800, Cat#234-004, Synaptic systems, Goettingen, Germany)-validation information on company website, staining profile was compared with the Rabbit anti-Iba-1 to confirm specificity;<br>Rabbit anti-P2Y12R (1:500, Cat#AS-55043A, Anaspec, Fremont, CA)-validation information on company website, used within recommended concentration, staining compared with Iba-1 in uninjured mouse brain tissue to confirm specificity for microglia;<br>Rabbit anti-mouse IgG (1:1000, Cat#97042, Abcam, Cambridge, MA)-validation information on manufacturers website and used at the recommended concentration for IHC by the manufacturer;<br>Rat anti-Ly6B2 (1:200, Cat#MCA771GT, Bio-Rad, Hercules, CA)-validation information on manufacturers website and used at the recommended concentration for IHC, used by us previously in publications;<br>Goat anti-PDGFR-β (1:200, Cat#AF1042, R&D systems, Minneapolis, MN)-validation information on manufacturers website and used at the recommended concentration for IHC;<br>Rabbit anti-Olig2 (1:200, Cat#AB9610, Millipore, Burlington, MA)-validation information on manufacturers website and used at the recommended concentration for IHC.

Animals and other organisms

Policy information about studies involving animals; ARRIVE guidelines recommended for reporting animal research

Laboratory animals	See methods section, page 29 under the section heading "Animals". Across all experiments wildtype or transgenic C57/BL6 female and male mice that were aged between 8 weeks and four months old at the time of craniotomy surgery were used. Mice were housed in a 12-hour light/dark cycle in a specific pathogen-free facility with controlled temperature (maintained within range of 20-26°C) and humidity (maintained within range of 30-70%) and were provided with food and water ad libitum.
Wild animals	No wild animals were used in the study.
Field-collected samples	No field collected samples were used in the study.
Ethics oversight	See methods section, page 23 under the section heading "Animals". All in vivo experiments involving the use of mice were conducted according to protocols approved by the Animal Research Committee (ARC) of the Office for Protection of Research Subjects at University of California Los Angeles (UCLA).

Note that full information on the approval of the study protocol must also be provided in the manuscript.
